# 

**Published:** 1908-06-15

**Authors:** 


					﻿Dental
Register
NELVILLE S. HOFF, Editor,
Ann Arbor, Mich.
Vol. LXI1	—JUNE, 1908— No. 6
SAMUEL A. CROCKER & CO., Publishers
OHIO DENTAL AND SURGICAL DEPOT
35 W. FIFTH STREET, CINCINNATI, 0.
PRICE, ORE DOLLAR A YEAR
Entered at the Postoffice at Cincinnati, O., as second-class mail mattei
				

